# Supplementary material for: Overlapping cell population expression profiling and regulatory inference in C. elegans
Source: BMC Genomics. 2016 Feb 29;17:159. doi: 10.1186/s12864-016-2482-z (PMC4772325; doi:10.1186/s12864-016-2482-z)
Supplement: Additional file 13: — Web supplement. (DOC 21 kb) [file 12864_2016_2482_MOESM13_ESM.zip › sortWeb/clusters/hier.300.clusters/285.html]

Cluster 285 

## Cluster 285

### Expression

| cnd-1 rep. 1 | cnd-1 rep. 2 | cnd-1 rep. 3 | pha-4 rep. 1 | pha-4 rep. 2 | pha-4 rep. 3 | ceh-27 | ceh-36 | ceh-6 | F21D5.9 | mir-57 | mls-2 | pal-1 | pros-1 | ttx-3 | unc-130 | hlh-16 | irx-1 | ceh-6 (+) hlh-16 (+) | ceh-6 (+) hlh-16 (-) | ceh-6 (-) hlh-16 (+) | cnd-1 singlets | pha-4 singlets | 0 | 60 | 120 | 150 | 180 | 240 | 330 | 390 | 420 | 480 | 540 | 570 | 600 | 630 | 660 | NAME | Functional description |
| --- | --- | --- | --- | --- | --- | --- | --- | --- | --- | --- | --- | --- | --- | --- | --- | --- | --- | --- | --- | --- | --- | --- | --- | --- | --- | --- | --- | --- | --- | --- | --- | --- | --- | --- | --- | --- | --- | --- | --- |
|  |  |  |  |  |  |  |  |  |  |  |  |  |  |  |  |  |  |  |  |  |  |  |  |  |  |  |  |  |  |  |  |  |  |  |  |  |  | *comt-5* | Catechol-O-MethylTransferase family |
|  |  |  |  |  |  |  |  |  |  |  |  |  |  |  |  |  |  |  |  |  |  |  |  |  |  |  |  |  |  |  |  |  |  |  |  |  |  | *clec-86* | C-type LECtin |
|  |  |  |  |  |  |  |  |  |  |  |  |  |  |  |  |  |  |  |  |  |  |  |  |  |  |  |  |  |  |  |  |  |  |  |  |  |  | Y46D2A.1 |  |
|  |  |  |  |  |  |  |  |  |  |  |  |  |  |  |  |  |  |  |  |  |  |  |  |  |  |  |  |  |  |  |  |  |  |  |  |  |  | *srh-157* | Serpentine Receptor, class H |
|  |  |  |  |  |  |  |  |  |  |  |  |  |  |  |  |  |  |  |  |  |  |  |  |  |  |  |  |  |  |  |  |  |  |  |  |  |  | C01F6.2 |  |
|  |  |  |  |  |  |  |  |  |  |  |  |  |  |  |  |  |  |  |  |  |  |  |  |  |  |  |  |  |  |  |  |  |  |  |  |  |  | F35B12.10 |  |
|  |  |  |  |  |  |  |  |  |  |  |  |  |  |  |  |  |  |  |  |  |  |  |  |  |  |  |  |  |  |  |  |  |  |  |  |  |  | C36B7.1 |  |
|  |  |  |  |  |  |  |  |  |  |  |  |  |  |  |  |  |  |  |  |  |  |  |  |  |  |  |  |  |  |  |  |  |  |  |  |  |  | F45H7.13 |  |
|  |  |  |  |  |  |  |  |  |  |  |  |  |  |  |  |  |  |  |  |  |  |  |  |  |  |  |  |  |  |  |  |  |  |  |  |  |  | R07D5.9 |  |
|  |  |  |  |  |  |  |  |  |  |  |  |  |  |  |  |  |  |  |  |  |  |  |  |  |  |  |  |  |  |  |  |  |  |  |  |  |  | B0252.1 |  |
|  |  |  |  |  |  |  |  |  |  |  |  |  |  |  |  |  |  |  |  |  |  |  |  |  |  |  |  |  |  |  |  |  |  |  |  |  |  | C06H2.7 |  |
|  |  |  |  |  |  |  |  |  |  |  |  |  |  |  |  |  |  |  |  |  |  |  |  |  |  |  |  |  |  |  |  |  |  |  |  |  |  | F43C11.11 |  |
|  |  |  |  |  |  |  |  |  |  |  |  |  |  |  |  |  |  |  |  |  |  |  |  |  |  |  |  |  |  |  |  |  |  |  |  |  |  | *ins-10* | INSulin related |
|  |  |  |  |  |  |  |  |  |  |  |  |  |  |  |  |  |  |  |  |  |  |  |  |  |  |  |  |  |  |  |  |  |  |  |  |  |  | R02C2.7 |  |
|  |  |  |  |  |  |  |  |  |  |  |  |  |  |  |  |  |  |  |  |  |  |  |  |  |  |  |  |  |  |  |  |  |  |  |  |  |  | Y38E10A.2 |  |
|  |  |  |  |  |  |  |  |  |  |  |  |  |  |  |  |  |  |  |  |  |  |  |  |  |  |  |  |  |  |  |  |  |  |  |  |  |  | Y17D7C.2 |  |
|  |  |  |  |  |  |  |  |  |  |  |  |  |  |  |  |  |  |  |  |  |  |  |  |  |  |  |  |  |  |  |  |  |  |  |  |  |  | Y37D8A.27 |  |
|  |  |  |  |  |  |  |  |  |  |  |  |  |  |  |  |  |  |  |  |  |  |  |  |  |  |  |  |  |  |  |  |  |  |  |  |  |  | T05A1.16 |  |
|  |  |  |  |  |  |  |  |  |  |  |  |  |  |  |  |  |  |  |  |  |  |  |  |  |  |  |  |  |  |  |  |  |  |  |  |  |  | *srb-1* | Serpentine Receptor, class B (beta) |
|  |  |  |  |  |  |  |  |  |  |  |  |  |  |  |  |  |  |  |  |  |  |  |  |  |  |  |  |  |  |  |  |  |  |  |  |  |  | *srh-155* | Serpentine Receptor, class H |
|  |  |  |  |  |  |  |  |  |  |  |  |  |  |  |  |  |  |  |  |  |  |  |  |  |  |  |  |  |  |  |  |  |  |  |  |  |  | *sru-37* | Serpentine Receptor, class U |
|  |  |  |  |  |  |  |  |  |  |  |  |  |  |  |  |  |  |  |  |  |  |  |  |  |  |  |  |  |  |  |  |  |  |  |  |  |  | C14F11.22 |  |
|  |  |  |  |  |  |  |  |  |  |  |  |  |  |  |  |  |  |  |  |  |  |  |  |  |  |  |  |  |  |  |  |  |  |  |  |  |  | K03A1.9 |  |
|  |  |  |  |  |  |  |  |  |  |  |  |  |  |  |  |  |  |  |  |  |  |  |  |  |  |  |  |  |  |  |  |  |  |  |  |  |  | B0024.17 |  |
|  |  |  |  |  |  |  |  |  |  |  |  |  |  |  |  |  |  |  |  |  |  |  |  |  |  |  |  |  |  |  |  |  |  |  |  |  |  | F25D1.7 |  |
|  |  |  |  |  |  |  |  |  |  |  |  |  |  |  |  |  |  |  |  |  |  |  |  |  |  |  |  |  |  |  |  |  |  |  |  |  |  | C05B5.5 |  |
|  |  |  |  |  |  |  |  |  |  |  |  |  |  |  |  |  |  |  |  |  |  |  |  |  |  |  |  |  |  |  |  |  |  |  |  |  |  | *str-122* | Seven TM Receptor |
|  |  |  |  |  |  |  |  |  |  |  |  |  |  |  |  |  |  |  |  |  |  |  |  |  |  |  |  |  |  |  |  |  |  |  |  |  |  | C09E7.10 |  |
|  |  |  |  |  |  |  |  |  |  |  |  |  |  |  |  |  |  |  |  |  |  |  |  |  |  |  |  |  |  |  |  |  |  |  |  |  |  | *frpr-8* | FMRFamide Peptide Receptor family |
|  |  |  |  |  |  |  |  |  |  |  |  |  |  |  |  |  |  |  |  |  |  |  |  |  |  |  |  |  |  |  |  |  |  |  |  |  |  | C54C6.5 |  |
|  |  |  |  |  |  |  |  |  |  |  |  |  |  |  |  |  |  |  |  |  |  |  |  |  |  |  |  |  |  |  |  |  |  |  |  |  |  | *eat-4* | EATing: abnormal pharyngeal pumping |
|  |  |  |  |  |  |  |  |  |  |  |  |  |  |  |  |  |  |  |  |  |  |  |  |  |  |  |  |  |  |  |  |  |  |  |  |  |  | *tyra-2* | TYRAmine receptor |
|  |  |  |  |  |  |  |  |  |  |  |  |  |  |  |  |  |  |  |  |  |  |  |  |  |  |  |  |  |  |  |  |  |  |  |  |  |  | *npr-2* | NeuroPeptide Receptor family |
|  |  |  |  |  |  |  |  |  |  |  |  |  |  |  |  |  |  |  |  |  |  |  |  |  |  |  |  |  |  |  |  |  |  |  |  |  |  | ZK180.7 |  |
|  |  |  |  |  |  |  |  |  |  |  |  |  |  |  |  |  |  |  |  |  |  |  |  |  |  |  |  |  |  |  |  |  |  |  |  |  |  | *dop-1* | DOPamine receptor |
|  |  |  |  |  |  |  |  |  |  |  |  |  |  |  |  |  |  |  |  |  |  |  |  |  |  |  |  |  |  |  |  |  |  |  |  |  |  | *dct-12* | DAF-16/FOXO Controlled, germline Tumor affecting |
|  |  |  |  |  |  |  |  |  |  |  |  |  |  |  |  |  |  |  |  |  |  |  |  |  |  |  |  |  |  |  |  |  |  |  |  |  |  | *npr-19* | NeuroPeptide Receptor family |
|  |  |  |  |  |  |  |  |  |  |  |  |  |  |  |  |  |  |  |  |  |  |  |  |  |  |  |  |  |  |  |  |  |  |  |  |  |  | *flp-32* | FMRF-Like Peptide |
|  |  |  |  |  |  |  |  |  |  |  |  |  |  |  |  |  |  |  |  |  |  |  |  |  |  |  |  |  |  |  |  |  |  |  |  |  |  | *nep-11* | NEPrilysin metallopeptidase family |
|  |  |  |  |  |  |  |  |  |  |  |  |  |  |  |  |  |  |  |  |  |  |  |  |  |  |  |  |  |  |  |  |  |  |  |  |  |  | F58H1.7 |  |
|  |  |  |  |  |  |  |  |  |  |  |  |  |  |  |  |  |  |  |  |  |  |  |  |  |  |  |  |  |  |  |  |  |  |  |  |  |  | *ncx-5* | Na/Ca eXchangers |
|  |  |  |  |  |  |  |  |  |  |  |  |  |  |  |  |  |  |  |  |  |  |  |  |  |  |  |  |  |  |  |  |  |  |  |  |  |  | *twk-16* | TWiK family of potassium channels |
|  |  |  |  |  |  |  |  |  |  |  |  |  |  |  |  |  |  |  |  |  |  |  |  |  |  |  |  |  |  |  |  |  |  |  |  |  |  | *osta-2* | Organic Solute Transporter Alpha-like protein |
|  |  |  |  |  |  |  |  |  |  |  |  |  |  |  |  |  |  |  |  |  |  |  |  |  |  |  |  |  |  |  |  |  |  |  |  |  |  | *kcnl-1* | KCNN (potassium K ChaNNel, calcium activated)-Like |
|  |  |  |  |  |  |  |  |  |  |  |  |  |  |  |  |  |  |  |  |  |  |  |  |  |  |  |  |  |  |  |  |  |  |  |  |  |  | F59B2.13 |  |
|  |  |  |  |  |  |  |  |  |  |  |  |  |  |  |  |  |  |  |  |  |  |  |  |  |  |  |  |  |  |  |  |  |  |  |  |  |  | *tpra-1* | TPRA-1 (human TRansmembrane Protein, Adipocyte associated 1) ortholog |
|  |  |  |  |  |  |  |  |  |  |  |  |  |  |  |  |  |  |  |  |  |  |  |  |  |  |  |  |  |  |  |  |  |  |  |  |  |  | *klf-3* | Kruppel-Like Factor (zinc finger protein) |
|  |  |  |  |  |  |  |  |  |  |  |  |  |  |  |  |  |  |  |  |  |  |  |  |  |  |  |  |  |  |  |  |  |  |  |  |  |  | *cyp-33C3* | CYtochrome P450 family |

### Phenotypes enriched

none found

### Anatomy terms enriched

none found

### GO terms enriched

|  |  |  |
| --- | --- | --- |
| **GO term** | **Number of genes** | **FDR-corrected p-value** |
| integral component of membrane | 9 | 0.0075 |
| G-protein coupled receptor signaling pathway | 5 | 0.0140 |
| G-protein coupled receptor activity | 4 | 0.0240 |
| membrane | 9 | 0.0470 |

### Expression clusters enriched

|  |  |  |  |
| --- | --- | --- | --- |
| **Group name** | **Number in cluster** | **Enrichment** | **FDR corrected p** |
| Genes that show selective expression in a subset of cell types vs broadly expressed in many cell types. Correspond to 20% - 57% of enriched\_genes for a given cell type. WBPaper00037950:A-class-motor-neurons\_larva\_SelectivelyEnriched | 6 | 6.98 | 0.0448 |

### Motifs enriched

|  |  |  |  |  |  |
| --- | --- | --- | --- | --- | --- |
| **Motif** | **Logo** | **Possible orthologs** | **Number of motifs in cluster** | **Enrichment** | **FDR corrected p** |
| I$KR\_01 |  | ZK177.3 B0310.2 | 37 | 1.81 | 0.0003 |
| twi\_da\_SANGER\_5\_FBgn0003900 |  | hlh-15 hlh-32 hlh-8 | 33 | 1.88 | 0.0011 |
| Mv102 |  | nhr-6 (0.62) nhr-71 (0.52) nhr-2 nhr-213 nhr-68 | 40 | 1.60 | 0.0012 |
| V$MEF2\_03 |  | mef-2 | 20 | 2.80 | 0.0012 |
| V$TATA\_C |  | tbp-1 | 25 | 2.31 | 0.0014 |
| V$NKX25\_02 |  | ceh-24 (0.6) ceh-31 lim-7 lin-39 | 16 | 3.23 | 0.0022 |
| pTH9340 |  | mab-9 tbx-39 tbx-42 tbx-43 | 46 | 1.35 | 0.0025 |
| pTH9149 |  | ztf-30 | 39 | 1.58 | 0.0028 |
| Irx4\_2242 |  | irx-1 | 24 | 2.24 | 0.0034 |
| MA0201.1 |  | alr-1 ceh-36 pha-2 ceh-45 | 43 | 1.43 | 0.0035 |
| pTH4337 |  | crh-1 W08E12.1 | 33 | 1.77 | 0.0037 |
| V$S8\_01 |  | ceh-45 | 12 | 4.04 | 0.0037 |
| tgo\_sim\_SANGER\_5\_FBgn0015014 |  | hlh-30 aha-1 | 43 | 1.43 | 0.0038 |
| GM12878\_ETS1\_HudsonAlpha |  | odr-7 lin-1 slr-2 | 32 | 1.80 | 0.0039 |
| pTH10787 |  | alr-1 eyg-1 | 12 | 4.00 | 0.0040 |
| N$SKN1\_01 |  | skn-1 | 38 | 1.58 | 0.0045 |
| Vsx1\_1728 |  | alr-1 | 14 | 3.39 | 0.0046 |
| HLH1 |  | lin-32 hlh-11 hlh-15 hlh-1 | 33 | 1.74 | 0.0049 |
| pTH9245 |  | ceh-18 (0.66) | 25 | 2.10 | 0.0056 |
| Fer1\_da\_SANGER\_10\_FBgn0037475 |  | lin-32 ceh-32 lin-39 | 20 | 2.47 | 0.0057 |
| Six1\_0935 |  | ceh-32 | 41 | 1.47 | 0.0057 |
| HOXC10\_3 |  | php-3 lin-39 | 15 | 3.10 | 0.0059 |
| MA0502.1 |  | dro-1 ceh-20 | 19 | 2.55 | 0.0059 |
| pTH9163 |  | nhr-3 (0.57) | 39 | 1.53 | 0.0061 |
| MA0015.1 |  | che-1 | 43 | 1.41 | 0.0063 |
| CG14962\_SANGER\_5\_FBgn0035407 |  | C34H4.5 | 31 | 1.80 | 0.0063 |
| Pbx1\_3203 |  | ceh-20 | 35 | 1.65 | 0.0066 |
| TFE3\_1 |  | hlh-30 aha-1 | 41 | 1.46 | 0.0067 |
| pTH9974 |  | hlh-16 | 39 | 1.52 | 0.0068 |
| POU4F2\_1 |  | ceh-18 (0.66) unc-86 | 15 | 3.05 | 0.0068 |
| Jundm2\_0911 |  | skn-1 fos-1 | 16 | 2.88 | 0.0070 |
| I$MTTFA\_01 |  | hmg-5 | 46 | 1.30 | 0.0076 |
| V$CETS1P54\_02 |  | C52B9.2 | 23 | 2.17 | 0.0077 |
| pTH1739 |  | nhr-255 (0.52) | 48 | 1.22 | 0.0084 |
| NR2C1\_si |  | nhr-19 nhr-2 | 20 | 2.38 | 0.0087 |
| HXB6\_f1 |  | lin-39 | 30 | 1.78 | 0.0100 |
| ELF3\_f1 |  | C24A1.2 | 35 | 1.61 | 0.0100 |
| HXD9\_f1 |  | lin-39 | 37 | 1.55 | 0.0100 |
| Foxj3\_1 |  | lin-31 | 36 | 1.57 | 0.0110 |
| LHX2\_2 |  | egl-5 alr-1 ceh-14 ceh-43 ceh-2 lim-6 lim-7 lin-39 | 23 | 2.11 | 0.0110 |
| Lhx1\_2240 |  | lim-7 | 11 | 3.75 | 0.0120 |
| pTH6562 |  | ceh-5 | 27 | 1.88 | 0.0130 |
| pTH5690 |  | ceh-32 | 42 | 1.39 | 0.0140 |
| PHOX2B\_2 |  | alr-1 | 15 | 2.80 | 0.0150 |
| MA0260.1 |  | che-1 | 10 | 3.97 | 0.0150 |
| V$HMX1\_01 |  | ceh-9 | 31 | 1.70 | 0.0150 |
| MEIS1\_1 |  | ceh-32 | 47 | 1.24 | 0.0160 |
| K562\_SRF\_HudsonAlpha |  | unc-120 | 29 | 1.77 | 0.0160 |
| Irx3\_1 |  | irx-1 | 28 | 1.81 | 0.0160 |
| KLF3\_f1 |  | klf-1 klf-2 | 41 | 1.41 | 0.0160 |
| V$MYOD\_01 |  | ces-1 hlh-1 | 32 | 1.66 | 0.0170 |
| Ipf1\_3815 |  | ceh-53 ceh-45 lin-39 | 14 | 2.90 | 0.0170 |
| pTH9256 |  | ceh-18 (0.66) | 22 | 2.10 | 0.0170 |
| pTH10779 |  | nhr-182 | 20 | 2.23 | 0.0170 |
| FOXO1\_3 |  | daf-16 | 34 | 1.59 | 0.0180 |
| Tcf2\_0913 |  | hmbx-1 | 41 | 1.40 | 0.0180 |
| Mv75 |  | lin-31 elt-1 | 24 | 1.97 | 0.0180 |
| V$TCF11\_01 |  | skn-1 | 33 | 1.62 | 0.0190 |
| FEV\_f1 |  | lin-1 C24A1.2 | 41 | 1.40 | 0.0190 |
| Optix\_SOLEXA\_FBgn0025360 |  | ceh-34 | 21 | 2.14 | 0.0190 |
| pTH5539 |  | unc-120 | 30 | 1.71 | 0.0200 |
| pTH9297 |  | ceh-18 (0.66) | 22 | 2.07 | 0.0200 |
| Six6\_2267 |  | ceh-32 ceh-34 | 38 | 1.47 | 0.0210 |
| pTH5018 |  | fos-1 atf-7 | 29 | 1.74 | 0.0210 |
| EN1\_2 |  | ceh-16 | 7 | 5.53 | 0.0210 |
| GRHL1\_2 |  | grh-1 | 28 | 1.77 | 0.0210 |
| pTH3998 |  | tbx-39 | 32 | 1.64 | 0.0210 |
| pTH10041 |  | ztf-29 | 33 | 1.60 | 0.0220 |
| pTH9211 |  | C01B12.2 attf-1 | 21 | 2.11 | 0.0220 |
| HepG2\_FOXA2\_HudsonAlpha |  | let-381 | 48 | 1.19 | 0.0220 |
| pTH9244 |  | tbx-39 | 16 | 2.54 | 0.0220 |
| pTH9907 |  | nhr-34 | 37 | 1.49 | 0.0230 |
| MA0456.1 |  | ref-2 | 38 | 1.46 | 0.0230 |
| MSX1\_1 |  | ceh-43 ceh-1 lin-39 | 12 | 3.14 | 0.0240 |
| Barx1\_2877 |  | ceh-43 | 27 | 1.79 | 0.0240 |
| pTH9381 |  | ceh-18 (0.66) | 35 | 1.53 | 0.0250 |
| pTH10721 |  | ceh-18 (0.66) ztf-9 | 27 | 1.79 | 0.0250 |
| SOX10\_1 |  | sox-4 | 44 | 1.31 | 0.0250 |
| pTH10630 |  | lsy-27 | 33 | 1.59 | 0.0260 |
| Pou3f1\_3819 |  | ceh-6 | 35 | 1.53 | 0.0260 |
| STF1\_f1 |  | nhr-68 | 41 | 1.38 | 0.0260 |
| V$NCX\_01 |  | ceh-19 | 29 | 1.71 | 0.0260 |
| SPDEF\_1 |  | lin-1 C24A1.2 | 30 | 1.67 | 0.0270 |
| pTH5423 |  | klf-2 | 42 | 1.35 | 0.0270 |
| pTH5976 |  | irx-1 | 44 | 1.30 | 0.0270 |
| Barx2\_3447 |  | ceh-43 | 31 | 1.63 | 0.0290 |
| Spdef\_0905 |  | lin-1 | 42 | 1.35 | 0.0290 |
| pTH8330 |  | C34D1.1 | 22 | 2.00 | 0.0300 |
| pTH5877 |  | nhr-100 | 34 | 1.55 | 0.0300 |
| MA0142.1 |  | ceh-6 | 34 | 1.54 | 0.0300 |
| Hoxd11\_3873 |  | php-3 | 37 | 1.47 | 0.0300 |
| ELK3\_1 |  | lin-1 | 33 | 1.57 | 0.0310 |
| MA0046.1 |  | ceh-53 hmbx-1 | 12 | 3.01 | 0.0320 |
| MA0131.1 |  | F39B2.1 | 42 | 1.34 | 0.0320 |
| Six4\_2860 |  | ceh-32 | 14 | 2.67 | 0.0320 |
| V$VMYB\_01 |  | D1081.8 | 19 | 2.17 | 0.0320 |
| TBX3\_f1 |  | tbx-39 | 36 | 1.49 | 0.0330 |
| Nkx1-2\_3214 |  | ceh-30 | 28 | 1.72 | 0.0340 |
| Blimp-1\_SANGER\_5\_FBgn0035625 |  | blmp-1 | 12 | 2.98 | 0.0340 |
| OLIG3\_1 |  | hlh-32 ngn-1 | 44 | 1.29 | 0.0340 |
| Nkx1-1\_3856 |  | ceh-30 | 28 | 1.71 | 0.0340 |
| EN2\_1 |  | ceh-31 ceh-16 | 9 | 3.80 | 0.0350 |
| pTH3819 |  | ceh-18 (0.66) | 19 | 2.15 | 0.0350 |
| pTH4325 |  | ceh-18 (0.66) | 25 | 1.82 | 0.0360 |
| pTH9026 |  | attf-1 | 16 | 2.39 | 0.0360 |
| NR2F6\_f1 |  | nhr-2 | 29 | 1.67 | 0.0370 |
| FOXO4\_1 |  | daf-16 | 39 | 1.40 | 0.0370 |
| pTH6268 |  | ceh-2 | 19 | 2.14 | 0.0370 |
| Tbp\_pr781 |  | tbp-1 | 11 | 3.15 | 0.0370 |
| Sox1\_4 |  | sox-4 | 35 | 1.50 | 0.0380 |
| Tin\_SOLEXA\_FBgn0004110 |  | ceh-22 | 45 | 1.26 | 0.0380 |
| pTH5083 |  | fos-1 crh-1 | 30 | 1.63 | 0.0390 |
| pTH6071 |  | C33G8.2 | 44 | 1.28 | 0.0390 |
| MA0043.1 |  | ces-2 | 32 | 1.57 | 0.0410 |
| Gmeb1\_1745 |  | attf-1 | 12 | 2.89 | 0.0410 |
| pTH3751 |  | tbx-39 | 19 | 2.11 | 0.0420 |
| pTH10024 |  | F52B5.7 | 41 | 1.35 | 0.0430 |
| pTH6449 |  | ceh-43 | 29 | 1.65 | 0.0440 |
| pTH10638 |  | dmd-3 | 43 | 1.30 | 0.0440 |
| pTH0977 |  | klf-1 | 40 | 1.37 | 0.0450 |
| pTH5437 |  | ceh-34 | 40 | 1.37 | 0.0450 |
| HNF6\_f1 |  | ceh-48 | 9 | 3.61 | 0.0460 |
| Hlxb9\_3422 |  | ceh-12 | 31 | 1.59 | 0.0460 |
| pTH6445 |  | ceh-5 | 23 | 1.87 | 0.0460 |
| pTH6508 |  | nhr-36 | 45 | 1.25 | 0.0460 |
| pTH8745 |  | attf-1 | 12 | 2.84 | 0.0470 |
| Spdef |  | lin-1 | 43 | 1.30 | 0.0470 |
| V$ROAZ\_01 |  | C09F5.3 | 34 | 1.50 | 0.0470 |
| Lbx2\_3869 |  | mls-2 | 42 | 1.32 | 0.0490 |
| Hoxa11\_2218 |  | php-3 | 33 | 1.52 | 0.0500 |
| pTH5891 |  | nhr-49 | 14 | 2.51 | 0.0500 |
| PRRX1\_f1 |  | alr-1 | 5 | 6.82 | 0.0500 |

### Correlated (and anti-correlated) transcription factors

|  |  |
| --- | --- |
| **Transcription factor** | **Correlation** |
| nhr-1 | 0.83 |
| med-2 | 0.77 |
| ctbp-1 | 0.76 |
| tag-97 | 0.75 |
| mbr-1 | 0.74 |
| egl-13 | 0.72 |
| ceh-17 | 0.70 |
| daf-3 | 0.69 |
| crh-2 | 0.68 |
| madf-4 | 0.67 |
| zip-1 | 0.66 |
| ceh-18 | 0.66 |
| lfi-1 | 0.65 |
| hlh-34 | 0.64 |
| sem-4 | 0.64 |
| zfh-2 | 0.64 |
| ceh-62 | 0.64 |
| nhr-20 | 0.63 |
| nhr-40 | 0.63 |
| F13H6.1 | 0.62 |
| nhr-6 | 0.62 |
| ZC328.2 | 0.62 |
| C34D10.2 | 0.61 |
| tag-68 | 0.61 |
| F26A10.2 | 0.61 |
| dhhc-13 | -0.41 |
| Y53F4B.3 | -0.41 |
| tbx-41 | -0.41 |
| nhr-229 | -0.42 |
| hlh-29 | -0.42 |
| ztf-4 | -0.42 |
| sup-35 | -0.42 |
| ces-1 | -0.43 |
| sma-4 | -0.43 |
| nhr-13 | -0.43 |
| ztf-13 | -0.44 |
| ceh-40 | -0.45 |
| C01F6.9 | -0.45 |
| repo-1 | -0.46 |
| C16A3.4 | -0.47 |
| hmg-11 | -0.47 |
| Y48G9A.11 | -0.47 |
| hmg-5 | -0.48 |
| cep-1 | -0.49 |
| mxl-1 | -0.49 |
| hmg-6 | -0.50 |
| Y56A3A.18 | -0.51 |
| zip-7 | -0.52 |
| D2030.7 | -0.55 |
| cebp-2 | -0.59 |

### ChIP peaks enriched

none found
